# Ultrasound-Guided Popliteal Nerve Block with Short-Acting Lidocaine in the Surgical Treatment of Ingrown Toenails

**DOI:** 10.3390/ijerph18105059

**Published:** 2021-05-11

**Authors:** Beom Suk Kim, Kyungho Kim, Jonathan Day, Jesse Seilern Und Aspang, Jaeyoung Kim

**Affiliations:** 1 Uijeongbu Eulji Medical Center, Department of Physical Medicine and Rehabilitation, Eulji University, Daejeon 11759, Korea; mattkim9966@gmail.com; 2 Department of Physical Medicine and Rehabilitation, Korea University College of Medicine, Seoul 02841, Korea; 3 Department of Orthopedic Surgery, Armed Forces Daejeon Hospital, Daejeon 34059, Korea; keonghos@gmail.com; 4 Samsung Medical Center, Department of Orthopedic Surgery, Seoul 06351, Korea; 5 Department of Orthopedic Surgery, Hospital for Special Surgery, New York, NY 10021, USA; jd1553@georgetown.edu (J.D.); jesseseilern@gmail.com (J.S.U.A.); 6 School of Medicine, Georgetown University, Washington, DC 20007, USA; 7 Department of Orthopedic Surgery, Emory University School of Medicine, Atlanta, GA 30322, USA

**Keywords:** ingrown toenail, digital block, popliteal block, ultrasound, sciatic nerve, anesthesia

## Abstract

Background: Digital nerve block (DB) is a commonly utilized anesthetic procedure in ingrown toenail surgery. However, severe procedure-related pain has been reported. Although the popliteal sciatic nerve block (PB) is widely accepted in foot and ankle surgery, its use in ingrown toenail surgery has not been reported. Therefore, this study aimed to investigate the safety and effectiveness of PB in the surgical treatment of ingrown toenails. Methods: One-hundred-ten patients surgically treated for an ingrown toenail were enrolled. Sixty-six patients underwent DB, and 44 underwent PB. PB was performed under ultrasound-guidance via a 22-gauge needle with 15 mL of 1% lidocaine in the popliteal region. The visual analogue scale was used to assess pain at two-time points: pain with skin penetration and pain with the solution injection. Time to sensory block, duration of sensory block, need for additional injections, and adverse events were recorded. Results: PB group demonstrated significantly lower procedure-related pain than the DB group. Time to sensory block was significantly longer in the PB group (20.8 ± 4.6 versus 6.5 ± 1.6 minutes). The sensory block duration was significantly longer in the PB group (187.9 ± 22.0 versus 106.5 ± 19.1 minutes). Additional injections were required in 16 (24.2%) DB cases, while no additional injections were required in PB cases. Four adverse events occurred in the DB group and two in the PB group. Conclusion: PB was a less painful anesthetic procedure associated with a longer sensory block duration and fewer repeat injections compared with DB. The result of this study implicates that PB can be an alternative anesthetic option in the surgical treatment of ingrown toenails.

## 1. Background

An ingrown toenail is one of the most common painful conditions affecting the nail of the great toe. Surgical treatments are warranted when conservative treatment is not effective [1,2,3,4].

In the surgical treatment of ingrown toenails, digital nerve block (DB) has been the most frequently used anesthetic technique due to its simplicity and rapid onset time [2,5]. However, considerable procedure-related pain and discomfort have been reported in DB, which cause considerable stress and anxiety to patients [6,7,8,9,10]. Moreover, two injections are often required in both medial and lateral sides of the nail fold. Despite these shortcomings, no other anesthetic methods have been examined in the surgical treatment of ingrown toenails.

Popliteal sciatic nerve block (PB) has been widely used in the field of foot and ankle surgery with minimal risk and excellent patient satisfaction [11,12]. With recent advances in technology, ultrasound (US) enabled clinicians to accurately and reliably evaluate anatomic structures [13], and to safely perform injections under the US-guidance. When performing PB, clinicians should first identify the tibial nerve at the popliteal crease. The tibial nerve can be easily identified just lateral and superficial to the popliteal artery, then tracing proximally along its course should be followed to find the bifurcation point of the sciatic nerve where the tibial nerve merge with the peroneal nerve. The PB is performed just proximal to the bifurcation point [14,15]. Moreover, it is possible to achieve anesthesia of both nail folds with a single injection if bilateral nail folds are involved. Despite these potential advantages, their use in ingrown toenail surgery is novel and has not been previously described.

This study aimed to investigate the safety and effectiveness of PB with short-acting lidocaine in the surgical treatment of ingrown toenails compared to conventional DB. The current study hypothesized that the PB would be a safe and effective anesthetic method with lower procedure-related pain in ingrown toenail surgery.

## 2. Methods

### 2.1. Subjects

This study was conducted in the armed forces hospital and approved by the Institutional Review Board of the medical command. Informed consent was obtained from all of the patients. The medical records of 150 patients who underwent surgical treatment of ingrown toenail between 2016 and 2017 were reviewed. Patients who underwent solitary ingrown toenail surgery were included in the study cohort. Patients who (1) underwent other types of foot and ankle procedures at the time of ingrown toenail surgery and (2) used anesthetic agents other than lidocaine were excluded from the study.

Ultimately, a total of 110 patients constituted our cohort. Patients were divided into two groups, according to the anesthetic method used for the surgical treatment; popliteal block (PB, *n* = 44) group and digital block (DB, *n* = 66) group. The mean age of the PB group was 20.7 years (range, 18–36), and the mean body mass index (BMI) was 23.6 kg/m^2^ (range, 20.3–31.3). The mean age of the DB group was 20.9 years (range, 19–31), and the mean body mass index (BMI) was 24.6 kg/m^2^ (range, 20.3–31.3). The demographic characteristics of the study groups are tabulated in Table 1.

Surgical treatment of ingrown toenails was performed in patients with Stage 2 or 3 according to the Heifetz classification: Stage 1, slight erythema and swelling of the nail grooves in the nail bed; Stage 2, presence of acute infection and suppuration; Stage 3, chronic infection with formation of granulation tissue around the nail groove and hypertrophy of surrounding tissues [16]. All patients who underwent surgical treatment were initially managed with conservative treatment, including oral antibiotic agents.

### 2.2. Anesthetic Method and Technique

The choice of anesthesia method was determined during preoperative planning, in which the physician and patient discussed the benefits, risks, advantages, and disadvantages of DB and PB. Patients finally chose and consented to either DB or PB. The advantages of DB included the simplicity of the procedure and the fact that it has been most widely practiced [2]. The shortcomings of DB included pain associated with injection and the likelihood of multiple injections in some cases. The advantages of PB included safety and effectiveness in various other foot and ankle surgeries, as well as less pain associated with injections from patient experience [2,17,18,19]. The shortcomings of PB included the broader area of anesthesia, longer duration before wearing off, and not quite common use in the ingrown toenail.

All procedures including anesthesia and surgical treatment were performed by a single fellowship-trained orthopaedic surgeon with more than five years of experience in performing US-guided blocks.

**Figure 1:**
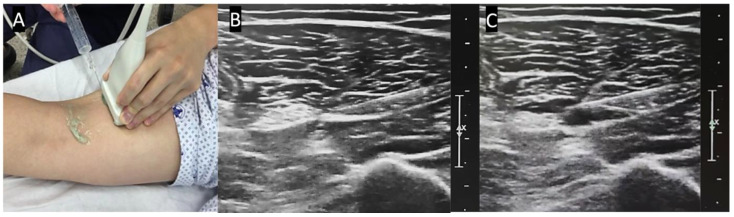
The popliteal block was performed under real-time ultrasound guidance. (**A**) From the popliteal crease, the tibial nerve was traced proximally along the posterior thigh. (**B**) The target of injection was chosen just proximal to the bifurcation point of the sciatic nerve. A needle was placed with a short-axis, in-plane, and lateral side approach to the sciatic nerve. (**C**) After confirming the needle tip position, subparaneural injection was performed with 15 mL of 1% lidocaine.

The PB was performed under US guidance. The US examinations were conducted with an HD 15 system (Philips Healthcare, Bothell, WA, USA) equipped with a 7–12 MHz linear transducer. Patients were positioned prone with the affected limb close to the practitioner. After povidone-iodine sterilization, the sciatic nerve was imaged in the short axis. Beginning from the popliteal crease, the tibial nerve was first identified just lateral and superficial to the popliteal artery, then traced proximally until it merged with the common peroneal nerve. The bifurcation point was identified where both branches (tibial and peroneal nerve) were situated contiguously [14,15]. The sciatic nerve was then traced proximally along the posterior surface of the thigh. After a survey scan, the injection target was chosen just proximal to the bifurcation point of the sciatic nerve. A 22-gauge, 70-mm spinal needle was placed with a short-axis, in-plane, and lateral side approach to the sciatic nerve. After confirming the needle tip position, subparaneural injection was performed with 15 mL of 1% lidocaine solution (7.5 mL of 2% lidocaine mixed with 7.5 mL of normal saline). The local anesthetic solution was administered inside the paraneural sheath, but not into the nerve bundles. The needle tip position was adjusted as necessary to ensure the subparaneural spread of the solution around the sciatic nerve (Figure 1). It mostly took less than 5 minutes to identify the target nerve and to complete the injection procedure.

The DB was performed with patients positioned supine. After povidone-iodine sterilization, a 25-gauge needle was introduced into the dorsal aspect of the webspace aiming towards the dorsal digital nerve just distal to the metatarsophalangeal joint. Then, 1.5 ml of 2% lidocaine was administered. The needle was withdrawn and redirected to the toe’s plantar surface, where the remaining solution (1.5 mL) was injected towards the plantar digital nerve. The procedure was repeated similarly on the opposite side of the toe to achieve sufficient anesthesia.

### 2.3. Surgical Technique

All patients involved in this study underwent partial nail extraction with matrixectomy of the hallux. After sufficient anesthesia was achieved, a distal skin incision was made over the nail sulci and then advanced to the proximal nail base. An excision of embedded part of the nail was performed. The germinal matrix was curetted and coagulated with an electrocautery device (ConMed Corp, Utica, NY, USA) (power 50v), then sutured with 4-0 nylon. The compressive dressing was applied, and stitches were removed two weeks postoperatively.

### 2.4. Outcome Measures

The primary outcome measure was procedure-related pain intensity at two different time points: pain during skin penetration and pain during the solution injection. This was assessed using the visual analog scale (VAS, mm) ranging from 0 to 10 mm, with a greater score indicating more severe pain. Secondary outcome measures were block characteristics of the two anesthetic methods: time to sensory block, duration of sensory block, additional injections required due to insufficient anesthesia, and adverse reactions to anesthesia (e.g., nausea, vomiting, dizziness). Once the anesthetic procedure was complete, an independent observer assessed block progression every 3 minutes for 30 minutes until the sensory block was achieved. Sensory examination was performed by the pinprick test of both the tibial and common peroneal distributions (plantar and dorsal aspects of the distal phalanx, respectively). It was assumed that the sensory block was achieved when there was no response to the pinprick stimulus. After the operation, the same observer evaluated the duration of the block, which was the time between the sensory block achievement and the point when the patient started to feel pain. All subjects were observed in the hospital for at least 4 hours after anesthesia for potential adverse reactions, such as nausea, vomiting, or dizziness.

### 2.5. Statistical Analysis

Data analyses were conducted using Prism 8 for Mac (Graphpad, CA, USA). The assumption of normality in data was tested using the Shapiro–Wilk test. An independent t-test was used to compare procedures-related pain, time to sensory block, and duration of the sensory block between the two groups (DB and PB). Chi-square analysis was used to compare differences in the needs for additional injections due to insufficient anesthesia and adverse events related to anesthesia. *P*-values less than 0.05 were considered statistically significant.

## 3. Results

**Figure 2:**
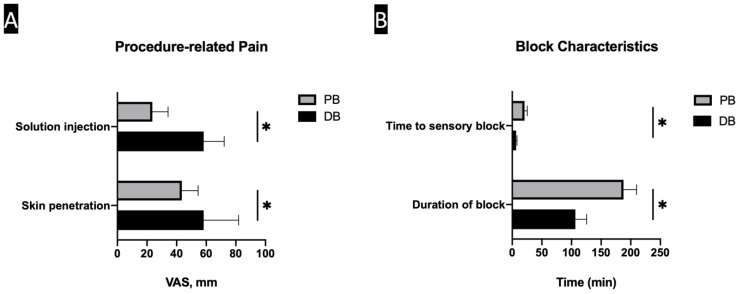
Bar graphs showing the Procedure-related pain (**A**) and block characteristics (**B**) in two groups. (**A**) The popliteal sciatic nerve block (PB) group showed significantly lower procedure-related pain than the digital nerve block (DB) group. (**B**) Time to sensory block and the duration of sensory block was significantly longer in the PB group. * statistically significant (*p* < 0.001).

The PB group showed significantly lower procedure-related pain than the DB group (*p* < 0.001). The mean VAS scores during skin penetration were 43.5 mm (SD, ±11.0) and 58.3 mm (±23.6) in PB and DB, respectively. The mean VAS scores during the injection of solution were 23.6 mm (±10.6) and 58.3 mm (±13.8) in the PB and DB, respectively (Figure 2A).

The two anesthetic procedures showed different block characteristics. Time to sensory block and the duration of sensory block was significantly longer in the PB group (Figure 2B). Additional anesthetic injections were needed in 16 patients (24.2%) in the DB group due to inadequate anesthesia, while no additional injections were required in the PB group. A total of four adverse events (two patients developed dizziness and two developed syncope) occurred in the DB group, and two adverse events (one patient developed palpitations and one developed dizziness) happened in the PB group (Table 2).

## 4. Discussion

The PB demonstrated lower procedure-related pain, longer sensory block duration, and fewer repeat injections compared with DB. Besides, PB had a minimal number of adverse events, which was comparable to DB. We believe the result of this study implicates that PB can be a viable option in individual patients who are reluctant to undergo DB due to the pain associated with the procedure.

While DB has been the most commonly adopted anesthetic method in the surgical treatment of ingrown toenails, a considerable procedure-related pain has been reported. For this reason, there have been some clinical efforts to mitigate the pain associated with DB. Serour et al. evaluated the efficacy of topical EMLA cream (lidocaine 2.5% and prilocaine 2.5%) application on the area of injection before DB. However, they found no clinical benefit of topical cream compared to the placebo. The authors concluded that the topical anesthesia might not infiltrate to the deeply-located digital nerve where much of the pain is thought to originate from [20]. A study by Kose and colleagues reported a similar finding when topical alkaline vapocoolant spray’s effectiveness was evaluated. While the spray was found to relieve pain during initial skin penetration, VAS scores during injection of solution were not significantly decreased [21]. These studies suggest that the topical agents are not effective in relieving pain that is originated from the infiltration of the anesthetic agents.

In addition to procedure-related pain, DB often requires multiple injections, since both medial and lateral nail folds are frequently involved in the clinical presentation of ingrown toenails. Furthermore, as seen in this study, an additional injection of anesthetic agents is sometimes required to achieve full anesthesia in a toe with severely inflamed soft tissue due to its increased tissue acidity [22]. However, even when such additional injections are required, the use of a sufficient amount of anesthetic is limited, as large volumes of solutions can cause ischemia by pressure effect. Therefore, rather than modulating pain associated with DB, we focused on a different anatomic site of injection, of which the anesthesia can be achieved with a single injection while causing a lower level of procedure-related pain.

The nail folds are innervated by plantar digital nerves, which are branches from the medial and lateral plantar nerve, and the plantar nerves originates from the peroneal and tibial nerve [23]. Although this can also be accomplished by the ankle block method, it also requires multiple injections to achieve anesthesia both on peroneal and tibial nerves. Therefore, we thought the sciatic nerve in the popliteal region before bifurcation might be a reasonable anesthetic administration site that can accomplish anesthesia with a single injection.

The safety and effectiveness of the PB in the foot and ankle surgery have been well established in the literature [2,17,18,24]. US-guided technique enhances PB’s success rate since the sciatic nerve can be well visualized throughout the entire injection process in real-time [25]. Furthermore, as blood vessels are well identified on US and using Doppler mode enhances the visualization, the US-guidance minimizes the risk of vessel puncture. While DB essentially is performed without direct visualization of neurovascular structures, US-guided PB offers excellent visualization of underlying structures. Thus, there is theoretically less risk of iatrogenic injuries.

The pain at both skin penetration and anesthetic infiltration was lower in the PB group. We suspect that this may have been originated from the difference in the pain sensitivity between two target anatomic regions. Besides, due to the relatively small surface area of the toe, the pain caused by tissue expansion during solution injection is expected to be greater in DB.

In the current study, we utilized only short-acting lidocaine for PB because the surgical removal of the ingrown toenail is relatively a short procedure usually performed as a same-day outpatient surgery. In usual practice, a popliteal sciatic nerve block is completed with a mixture of rapid onset anesthetics such as lidocaine and more prolonged duration anesthetics such as ropivacaine or bupivacaine. The duration of anesthesia achieved by using this mixture is reported to be more than 10 hours [26,27]. Although we successfully obtained the level and duration of anesthesia required for our series, there is a utility in future studies investigating the minimal effective volume (MEV) of lidocaine necessary to obtain anesthesia in PB.

To our knowledge, this is the first study to date describing the use of PB in ingrown toenail surgery. Although PB demonstrated some advantages over DB in terms of procedure-related pain and block characteristics, the result of this study is not against or in favor of the substitution of DB. Instead, this study was designed to examine the efficacy of an alternative anesthetic method for surgical treatment of ingrown toenails. In our clinical experience, patients who had a bilateral ingrown toenail and had one side first with DB had greater reluctancy in receiving the same anesthetic method again due to pain from the anesthetic procedure. In those cases, PB can be considered as this can provide less pain related to the procedure.

The current study is not without limitations. First, this study was conducted in healthy males in their twenties. On average, this patient cohort’s health status may be different from the general population, which may explain for a relatively low adverse reaction rate to anesthesia. However, we believe the primary and secondary outcomes related to pain and block characteristics may be generalizable due to anatomic similarities. Another limitation is a relatively small sample size and retrospective nature of the study. However, we believe this preliminary investigation would provide a foundation for a future prospective randomized controlled trial comparing PB and DB in a larger cohort.

## 5. Conclusions

In conclusion, our data suggest that PB is a less painful anesthetic procedure with a longer duration of anesthesia and fewer repeat injections in the surgical treatment of ingrown toenails. The result of this study implicates that PB can be an alternative anesthetic option in the surgical treatment of ingrown toenails for patients who are reluctant to undergo DB.

## Figures and Tables

**Table 1:** Demographic characteristics of the study groups ^a^.

	PB Group (*n* = 44)	DB Group (*n* = 66)	*p*-Value
Male, *n* (%)	44 (100)	66 (100)	N/A
Age, years	20.7 ± 2.8	20.9 ± 2.0	0.663
BMI, kg/m^2^	23.6 ± 2.8	24.6 ± 3.0	0.082
Laterality, *n* (Right/Left)	18/26	34/32	0.331
Side of nail fold, *n* (Medial/Lateral/Both)	13/15/16	16/33/17	0.246

Abbreviations: PB, popliteal sciatic nerve block; DB, digital nerve block; BMI, body mass index; N/A, not available. ^a^: descriptive statistics are displayed as means and standard deviations for continuous variables and frequencies and percentages for categorical variables.

**Table 2:** Comparison of block characteristics and adverse events between groups ^a^.

Variables	PB Group (*n* = 44)	DB Group (*n* = 66)	*p*-Value
Time to sensory block, mean ± SD, min	20.8 ± 4.6	6.5 ± 1.6	< 0.01
Duration of sensory block, mean ± SD, min	187.9 ± 22.0	106.5 ± 19.1	< 0.01
Needs for additional injections, *n* (%)	0 (0)	16 (24.2)	< 0.01
Adverse events, *n* (%)	2 (4.5)	4 (6.1)	0.732
Palpitation, *n* (%)	1 (2.3)	0 (0)	
Dizziness, *n* (%)	1 (2.3)	2 (3.0)	
Syncope, *n* (%)	0 (0)	2 (3.0)	

Abbreviations: PB, popliteal sciatic nerve block; DB, digital nerve block. ^a^ descriptive statistics are displayed as means and standard deviations for continuous variables and frequencies and percentages for categorical variables.

## Data Availability

The datasets used and analyzed during the current study are available from the corresponding author on reasonable request.

## Abbreviations

DB	Digital Nerve Block
PB	Popliteal Sciatic Nerve Block
US	Ultrasound
BMI	Body Mass Index
VAS	Visual Analogue Scale
MEV	Minimal Effective Volume
